# Comparative Study on the Performance of Five Different Hall Effect Devices

**DOI:** 10.3390/s130202093

**Published:** 2013-02-05

**Authors:** Maria-Alexandra Paun, Jean-Michel Sallese, Maher Kayal

**Affiliations:** STI-IEL-Electronics Laboratory, Ecole Polytechnique Fédérale de Lausanne (EPFL), CH-1015 Lausanne, Switzerland; E-Mails: jean-michel.sallese@epfl.ch (J.-M.S.); maher.kayal@epfl.ch (M.K.)

**Keywords:** Hall Effect sensor design, offset, sensitivity, device polarization, 3D physical simulations

## Abstract

Five different Hall Effect sensors were modeled and their performance evaluated using a three dimensional simulator. The physical structure of the implemented sensors reproduces a certain technological fabrication process. Hall voltage, absolute, current-related, voltage-related and power-related sensitivities were obtained for each sensor. The effect of artificial offset was also investigated for cross-like structures. The simulation procedure guides the designer in choosing the Hall cell optimum shape, dimensions and device polarization conditions that would allow the highest performance.

## 1. Introduction

One of the most commonly used sensing technologies today consists of CMOS Hall Effect sensors, based on magnetic phenomena. These sensors are primarily employed as current sensors and serve many low-power applications like position sensing and contactless switching within automotive and industrial electronics [1,2].

Regarding the Hall Effect device performance investigation, one would need to look, among others, for the highest sensitivity and the lowest offset. The geometry plays an important role in the Hall Effect sensors performance and was studied by the authors in [3–5]. A real Hall sensor has an offset due to geometrical errors, imperfections in the fabrication process, non-uniformity in material resistivity and thickness, *etc.* [6].

The offset and sensitivity are important figures of merit in Hall sensors performance evaluation [7]. Within the electronic circuit, the offset can be reduced by current-spinning methods. In addition to this, a well chosen geometry of the Hall device itself can greatly minimize the resulting value. By using the dynamic offset cancelation technique proposed in [8] the offset was kept below 10 μV without increasing too much the circuitry complexity. A few years later, the correlation of offset and geometry was analyzed by the authors. Four-phase residual offsets of certain implemented geometries were situated within maximum values of 2.5 μV at room temperature and therefore ensuring almost four times better performance than the state-of-the-art.

In the microelectronics recent development, the solving of fundamental semiconductor device equations by numerical methods is a productive investigation tool to predict the behavior and assess the performance of various devices.

The present paper analyzes the influence of the shape, dimensions, position of contacts and offset on the Hall Effect sensors performance, including Hall voltage and sensitivity, with the aid of three dimensional physical simulations. In this sense, the study also proposes an analysis of artificially induced offset. In order to ensure Hall Effect sensors optimal design, we use three-dimensional numerical solutions to the system of partial differential equations governing galvanomagnetic carrier transport in magnetic-field-sensitive semiconductors.

Section II presents the motivation behind the simulation approach, the basic physical model of the carrier transport in semiconductors and the methodology used for 3D structures simulation, presenting the design parameters for all analyzed Hall Effect devices. Section III is dedicated to accurate estimation of Hall voltage, different types of sensitivities and influence of geometrical mismatch on the structures offset by performing a comparative study on five different Hall devices. The purpose of this section is to finally reveal which of the simulated magnetic sensors displayed the best performance.

## 2. Methodology

### 2.1. Hall Effect Devices Integration and Analysis

In general, the Hall voltage is defined by the relation:

(1) $$V_{\text{HALL}}=G\frac{r_{H}}{\text{nqt}}I_{\text{bias}}B$$

where *G* is the geometrical correction factor, *r_H_* is the scattering factor of Silicon, usually 1.15, *n* is the carrier density, *t* is the thickness of the active region, *I_bias_* is the biasing current and *B* is the magnetic field induction [5].

The absolute sensitivity *S_A_* of a Hall sensor is given by the relation:

(2) $$S_{A}=\frac{V_{\text{HALL}}}{B}=G\frac{r_{H}}{\text{nqt}}I_{\text{bias}}$$

Relative sensitivities can also be defined. Therefore, the current-related *S_I_* and voltage-related *S_V_* sensitivities are introduced as follows:

(3) $$S_{I}=\frac{S_{A}}{I_{\text{bias}}};S_{V}=\frac{S_{A}}{V_{\text{bias}}}=\frac{S_{I}}{R}$$

where *V_bias_* is the bias voltage and *R* is the input resistance of the Hall device.

Since the Hall voltage and therefore sensitivity are inversely proportional to the n-well doping concentration, a lightly doped n-well is normally used in the fabrication process of the Hall Effect sensors.

Different Hall Effect devices were integrated in a 0.35 μm CMOS technology and evaluated for Hall voltage, sensitivity, offset, *etc.* More precisely eight different Hall cells were integrated and subsequently tested. A part of the experimental results in conjunction with the geometry influence analysis on the considered devices performance were presented in paper [3]. In order to analyze the sensors performance an automated AC measurement procedure was previously developed and an experimental data basis was created [5]. The objective was to design a certain Hall cell able to provide very small offsets less than 30 μT and their temperature drifts below 0.3 μT. The specified thresholds are already a few times better than state-of-the-art.

The offset analysis was of particular interest because in reality, Hall Effect sensors have offset. In this sense several samples, each one containing 64 cells (eight different geometries times eight locations), were tested. The experimental data obtained for the offset at room temperature for different biasing currents is presented in [5]. There is an influence of the particular device structure on the offset value.

Among the eight different integrated and tested Hall cells, the minimum offset was obtained for the XL, which is basically a classical Greek-cross but with the dimensions scaled up by a certain factor with respect to a basic shape. This particular geometry will be reproduced by simulation later within the present work, where additional details can be found including design parameters information.

**Figure 1.**
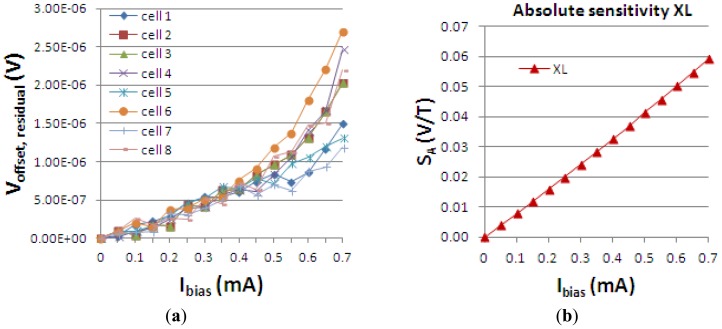
Measured 4-phases residual offset voltage *vs.* biasing current (**a**) and absolute sensitivity *vs.* biasing current (**b**) for XL cell.

Figure 1 presents both the measured 4-phases residual offset in V *vs. I_bias_* and the absolute sensitivity *vs. I_bias_* for the XL cell, tested 8 times using an automated measurement setup previously developed and presented by the authors in [5].

In particular, the residual offset mathematical function of the biasing current has a quadratic dependence, for the 2-phases spinning current [9]. The same quadratic increase of the residual offset with the biasing current is also observed by Demierre [10] in the Hall plate. In our case (Figure 1(a)), for 4-phases spinning current, as expected, the thermoelectric contribution proportional to *I_bias_*^2^ in the residual offset, cannot be completely compensated for *I_bias_* > 0.5 mA.

The aim of the present study is to employ three dimensional simulations for designing and selecting the best Hall device shape to be used in a certain integration process. The performance assessment is conducted by investigating the sensors Hall voltage, sensitivity and offset.

### 2.2. Hall Effect Devices Physical Simulation

In semiconductor materials, the classical carrier transport model [11–13] relies on the continuity equations. Moreover, we would also need to take into account the following partial differential equation in order to have a complete description of semiconductor physical behavior:

(4) −∇⋅(ε∇V)=q(p−n+N)

where *V* denotes the electrostatic potential, *ε* is the material electric permittivity, *q* is the electron charge and *N* = *N_D_* – *N_A_* is the fully ionized net impurity distribution. In turn, *n* and *p*, themselves functions of Fermi energy, temperature and electrostatic potential [4,11], represent electrons and holes densities, respectively. The solution of the Poisson's equation in (4) is the electrostatic potential *V*.

It is to be mentioned that the magnetic induction effect only manifests in the mathematical relations which define the current density. Equivalently speaking, in the absence of the magnetic field, the continuity equations and the Poisson's Equation (4) will remain the same.

By using the Synopsys Sentaurus TCAD tool [14], which solves the Poisson equation, both electrons and holes continuity equations, three-dimensional simulations of Hall sensors were performed. A 3D numerical modeling of carrier transport process in the magnetic field (electrostatic potential, current distributions) for semiconductor magnetic sensors with different geometries is used.

At each point of the grid, three unknowns will be considered, namely *V*, *n*, *p*. Further on, we would need three equations and the corresponding boundary conditions to solve the nonlinear system of partial differential equations. In order to have a correct solution, the discretization of the Poisson's equation, electron and holes continuity equations will be needed and a coupled method, which is a generalization of the Newton method, will be used to compute the initial proposed system by numerical iterative procedure.

The magnetic field acting on the semiconductor structure for Hall voltage generation was handled by the galvanic transport model. The analysis of magnetic field effects in semiconductor devices is done by solving the transport equation of electrons and holes inside the device. The usual drift-diffusion model of the carrier densities $\vec{{\text{J}}_{\text{n}}}$ and $\vec{{\text{J}}_{\text{p}}}$ should be rewritten by taking into account the magnetic field-dependent terms issued by the effect of Lorentz force on the carriers. Sentaurus includes the effect of magnetic field on semiconductors within the galvanic transport model. The following equation governs its behavior:

(5) $$\vec{J_{\alpha }}=\mu_{\alpha }\vec{g_{\alpha }}+\mu_{\alpha }\frac{1}{1+{\left({\mu_{\alpha }}^{*}B\right)}^{2}}\left[{\mu_{\alpha }}^{*}\vec{B}\times \vec{g_{\alpha }}+{\mu_{\alpha }}^{*}\vec{B}\times \left({\mu_{\alpha }}^{*}\vec{B}\times \vec{g_{\alpha }}\right)\right]$$

where *α* = *n*, *p*, $\vec{g_{\alpha }}$ is the current vector without mobility and μ_α_* is the Hall mobility [11].

The physical section of the simulation included a doping dependence mobility model together with Shockley-Read-Hall and Auger recombination processes as was considered in a paper [15]. In the actual context, it is assumed that the electrical ohmic contacts are ideal and the contact regions could support a sufficiently high dopant concentration. The electrostatic potential and carrier concentrations at the contact region are solved by the usual Dirichlet-type boundary conditions.

For a good tradeoff between accuracy and simulation run time, the three dimensional meshed structures of the Hall effect devices should contain a sufficient number of points. Smaller meshing dimensions and higher number of points increase the accuracy of the simulation results, but would require more CPU time and longer execution. With respect to the previous work the meshing strategy was improved.

The meshes of the simulated geometries contained between 40,000–70,000 points with refinement functions included to ensure maximum convergence and minimum numerical offset. Additional refinement windows have been placed on contacts in order to improve the simulation convergence and to decrease the numerical offset. A mesh step between 0.1 and 1 μm on the three axes was used for the mesh refinement window. In this way, for all structures the numerical offset does not exceed two milivolts for maximum biasing current. To address any further convergence issues, the magnetic field was ramped up to the required value of the magnetic induction.

### 2.3. Hall Effect Devices Modeling

To assess the Hall Effect sensors performance, FEM lumped circuit models were already developed by the authors [16]. The intention of the present paper is to reproduce from a physical point of view the shapes of the magnetic sensors that were already integrated in a CMOS technology and thoroughly tested by the authors [2,4]. To this purpose, five different three dimensional Hall Effect devices were modeled. The 3D simulation tool helps in modeling specific structures, while taking into account all the effects of carrier transport in semiconductors under magnetic field. The simulations results will provide useful information prior to integration in selecting potential Hall shapes with the best performance. In general these sensors are highly symmetric structures and invariant to an orthogonal rotation. Because any geometrical mismatch could significantly increase the offset, all the cells were accurately modeled. The analysis was focused on the classical Greek-cross with progressive dimensions increase (resulting in basic, L, XL cells), borderless and optimum cell. The implemented structures were intended to have the same fabrication process which is close to the one used when integrating the real Hall sensors. The Hall cells were all modeled on a Silicon p-substrate with an n-well active region.

Therefore, a p-substrate with 10^+15^ cm^−3^ boron concentration and an active n-well region doped with 1.5 × 10^+17^ cm^−3^ arsenic concentration in the form of a Gaussian profile implantation were used. This doping profile allows an average mobility of 0.0630 m^2^·V^−1^·s^−1^. The thickness is 5 μm for the p-substrate and 1 μm for the implantation of the n-doped profile active region, respectively.

Attention should be given to doping profile smoothing in order to ensure a good simulation convergence. Therefore the abrupt edges were avoided by imposing a decay length of a hundred of nanometers at the p-n junction. For testing purposes, each structure was endowed with four electrical contacts, amongst which two are for biasing the device and the other two opposite ones for the measurement of the voltage drop difference.

The geometrical design parameters of all the five simulated Hall Effect devices are given in Table 1. *L* and *W* stand for the cell length and width, respectively, while *s* represents the contact length. The width of the contacts is in general provided by the technology used in the Hall Effect devices fabrication process. In our simulations it was considered to be 0.7 μm. The distance from the contacts to the n-well borders is 0.35 μm for basic, L and XL cells, 13.85 μm for borderless cell, 5.5 μm for optimum cell. These values were omitted on the drawings for aesthetic purpose. The position of contacts with respect to borders is important in the offset analysis as contour errors might increase it.

**Figure 2.**
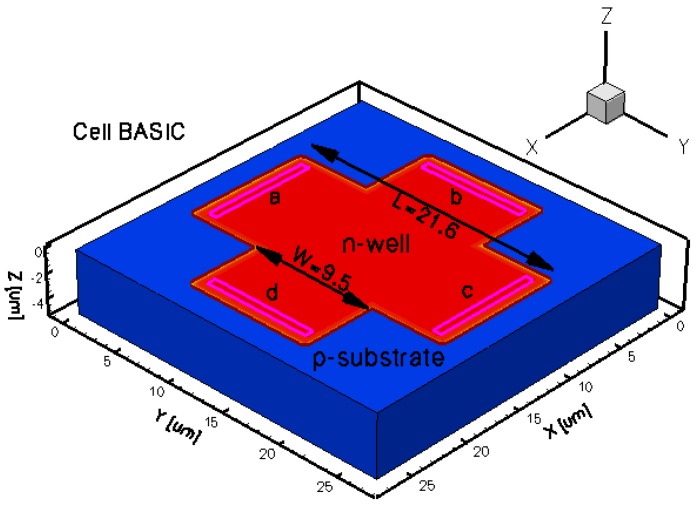
3D representation of the simulated basic Hall cell.

**Figure 3.**
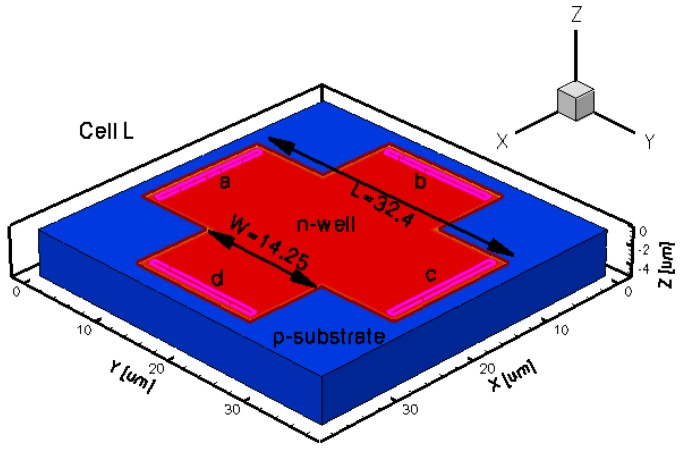
3D representation of the L simulated Hall cell.

**Figure 4.**
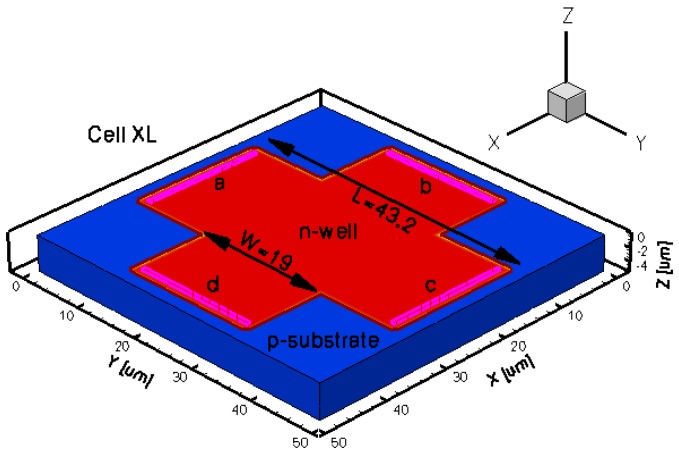
3D representation of the XL simulated Hall cell.

**Figure 5.**
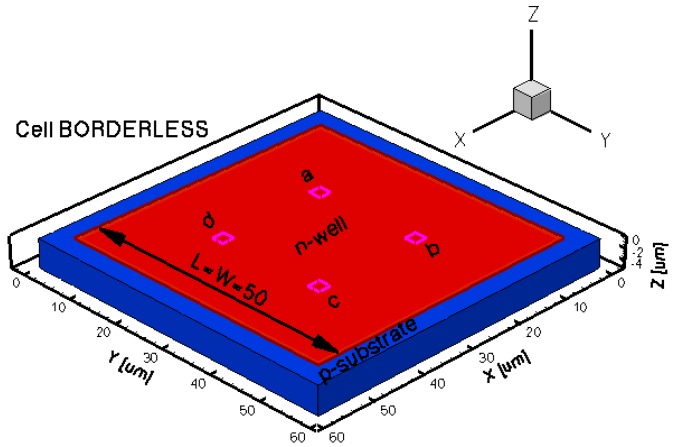
3D representation of the borderless simulated Hall cell.

**Figure 6.**
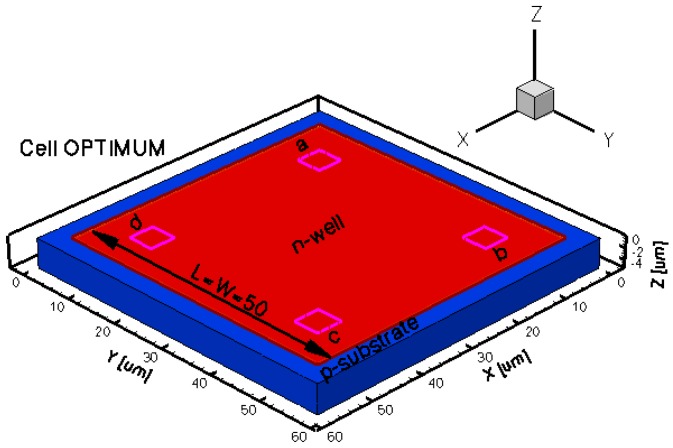
3D representation of the optimum simulated Hall cell.

The three-dimensional representations of the five simulated geometries are illustrated in Figures 2–6. The p-n junction is depicted by the line on the active region borders. The four electrical contacts (*a*-*d*) used for biasing and measurement purposes are depicted as well.

Regarding the polarization of Hall cells, imposing a certain voltage on electrode *a* will force a current to flow between contacts *a* and *c*. The Hall voltage is actually recorded as the voltage difference between the other two opposite contacts, *b* and *d* respectively. It is also to be mentioned that for the cross-like devices the current flows vertically from *a* to *c*, while for the borderless and optimum cell the circulation of current has a diagonal path, between *a* and *c* contacts.

## 3. Results and Discussion

For the analysis of the Hall Effect devices behavior, all the structures were simulated using current biasing, without and with magnetic field. In the present study, the biasing current was ramped from 0 to 1 mA.

The effects of the dimensions (input data), respectively the geometrical correction factor, on the Hall Effect sensors technical performance (output data) were analyzed by authors in a recent paper [3]. In fact, the shape and the distance from contacts to the p-n junction are important in the evaluation of the Hall Effect devices characteristics, including sensitivity and offset.

The classical cross structures dispose of contacts at the extremities of the four arms. The biasing and sensing will ensure a maximum sensitivity, but the structures can be more affected by any mismatch at the p-n junction. The idea is to increase the dimensions of this classical cross in order to be less prone to border asymmetries. The fourth analyzed shape, the borderless cell, is equipped with very small electrical contacts and they are located closer towards the center of the structure and farther away from the p-n junction. This specific structure could minimize the influence of any border errors but will also affect the sensitivity. The fifth shape, the optimum cell, is a combination of scaled up dimensions and contacts situated half way through with respect to the contacts of XL and borderless cells respectively.

### 3.1. Simulated Hall Cells I-V Characteristics and Resistance

**Figure 7.**
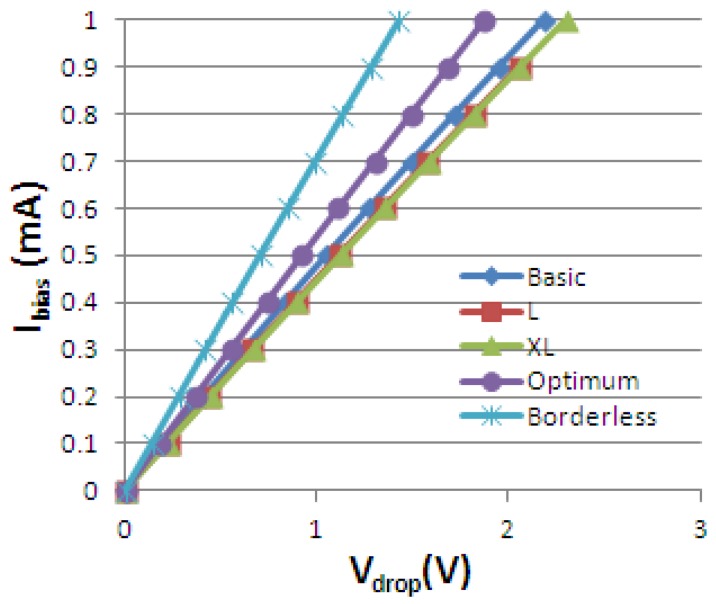
The simulated Hall devices I-V characteristics.

The Hall device I-V characteristic is obtained by simulations for each cell. Its representation for B = 0.5 T is incorporated in Figure 7. The resistance *R* for certain biasing currents is included in Table 2.

For the Hall Effect sensors, any nonlinearity that might be seen in the I-V characteristic is explained [6] by three possible mechanisms, such as material non-linearity, geometrical non-linearity and non-linearity due to the junction field effect. The material non-linearity and the geometrical non-linearity exhibit the same quadratic magnetic induction dependence, but have opposite signs, consideration which may be exploited to integrate Hall Effect sensors in which two non-linearity effects could cancel each other.

### 3.2. Simulated Hall Voltage, Electrostatic Potential and Conduction Current Density

The total output voltage of a Hall Effect device is given by the following relation:

(5) $$V_{\text{output}}=V_{\text{HALL}}+V_{\text{offset}}$$

Even though the shapes are symmetric we obtain a non-zero offset, which is the numerical offset from the simulator. Therefore, the meshing strategy was adapted to minimize it as much as possible.

We were interested to investigate the offset in order to have accurate information for Hall voltage and sensitivity, as the offset is a parasitic voltage adding to the total output voltage. The offset measurements were performed in the absence of magnetic field while for Hall voltage and sensitivity estimation the magnetic induction was considered B = 0.5 T. This particular value for the magnetic field induction was used to closely reproduce by simulation the magnetic field of B = 0.497 T used for the integrated Hall devices measurements.

**Figure 8.**
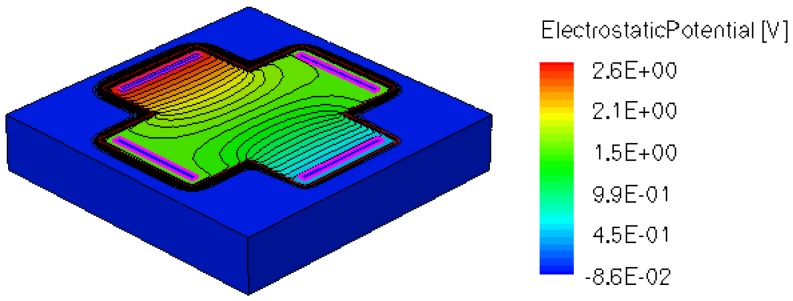
Electrostatic potential (V) for basic structure, B = 0.5 T.

**Figure 9.**
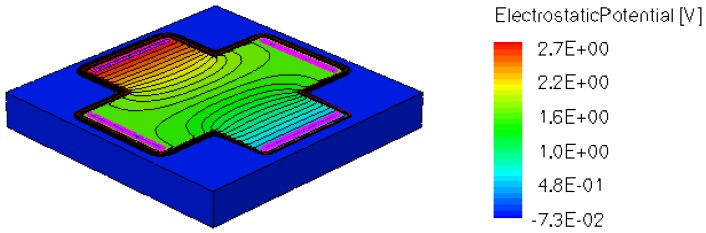
Electrostatic potential (V) for L structure, B = 0.5 T.

**Figure 10.**
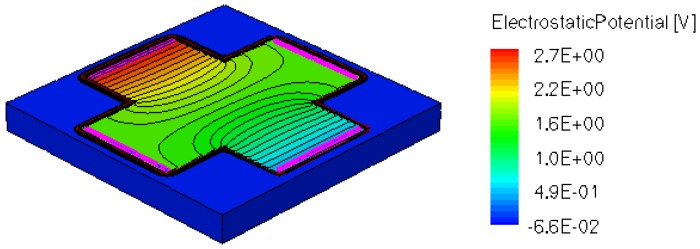
Electrostatic potential (V) for XL structure, B = 0.5 T.

**Figure 11.**
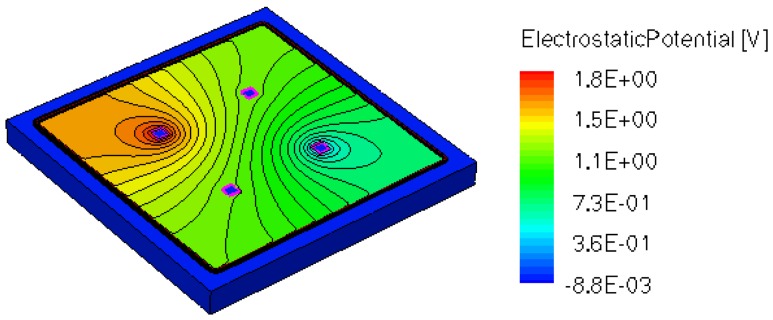
Electrostatic potential (V) for borderless structure, B = 0.5 T.

**Figure 12.**
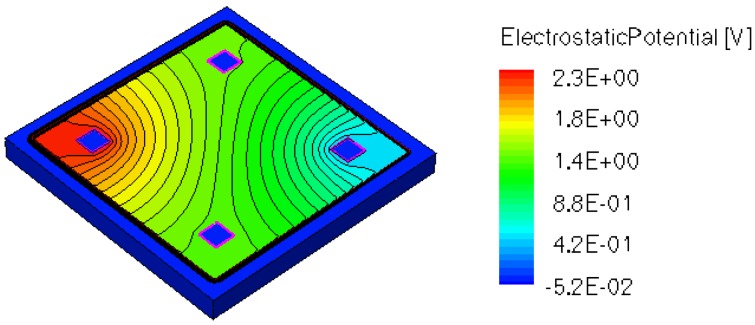
Electrostatic potential (V) for optimum structure, B = 0.5 T.

When applying a magnetic field of certain intensity, the carriers deviate under the influence of Lorentz force and thus the Hall voltage is forming between the opposite contacts. In Figures 8–12 we can see the three-dimensional structures of the simulated cells, basic, L, XL, optimum and borderless respectively, with the electrostatic potential distribution.

**Figure 13.**
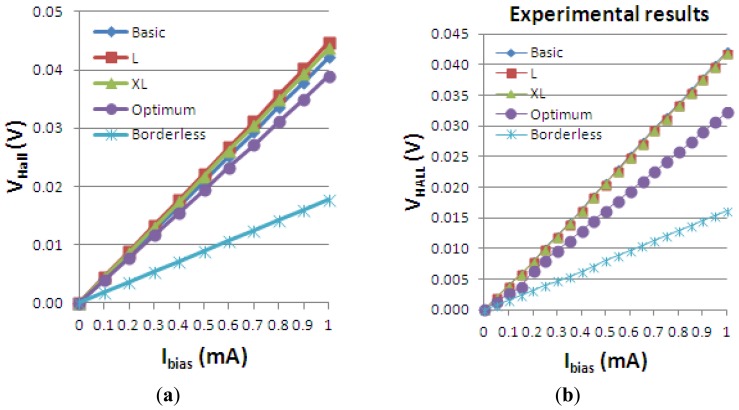
Hall Effect devices Hall voltage *vs. I_bias_*, as simulated (**a**) and as measured (**b**).

The Hall voltage of all the simulated structures is presented *vs.* biasing current in Figure 13(a). To assess the validity of the results obtained, we also added the measurement results for the five Hall cells in Figure 13(b). The cross-like cells have the same *L/W* ratio and they only differ by the scaling factor. Therefore, according to the definition of the Hall voltage in Equation (1), they are expected to have approximately the same *V_Hall_*. By investigating the obtained graph, the following numerical values are extracted. At maximum biasing current*, V_Hall_* = 42 mV for basic cell, *V_Hall_* = 44 mV for the L and XL cells, *V_Hall_* = 17.7 mV for the borderless cell and *V_Hall_* = 38 mV for the optimum cell, respectively.

**Figure 14.**
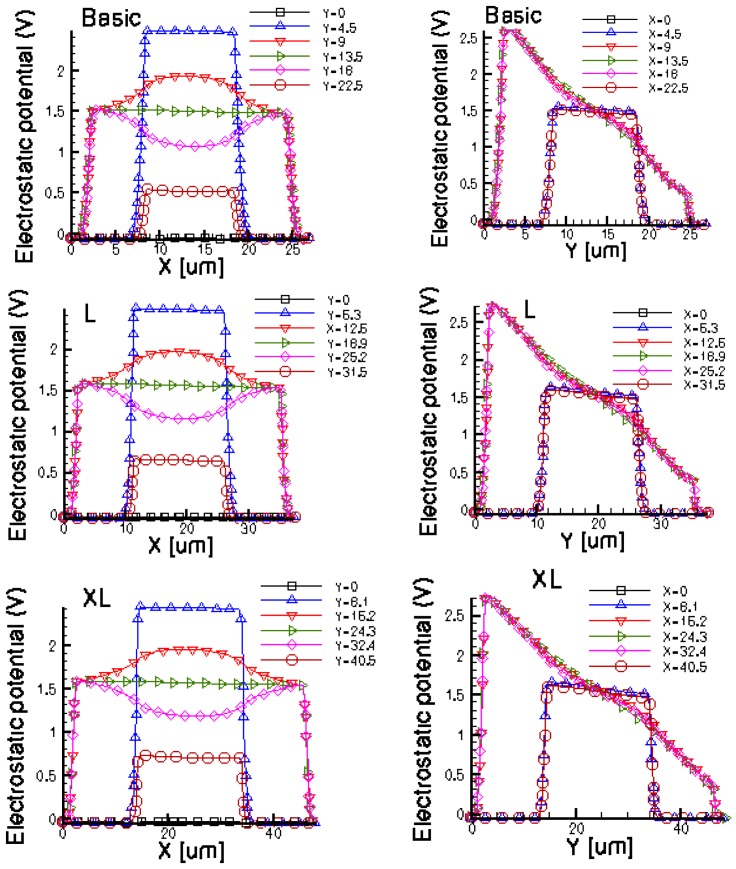
Electrostatic potential (V) in orthogonal cuts on Oy and Ox for the cross-like cells (XL, L and basic respectively).

**Figure 15.**
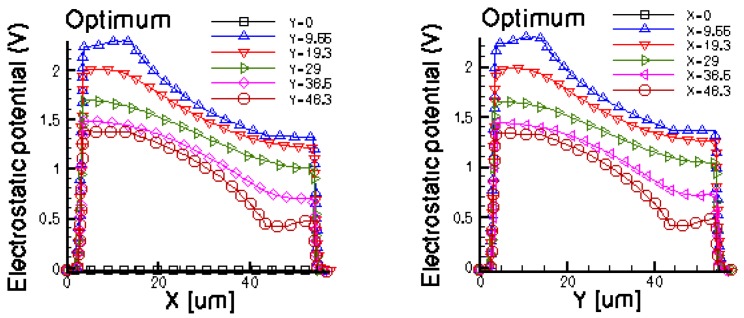

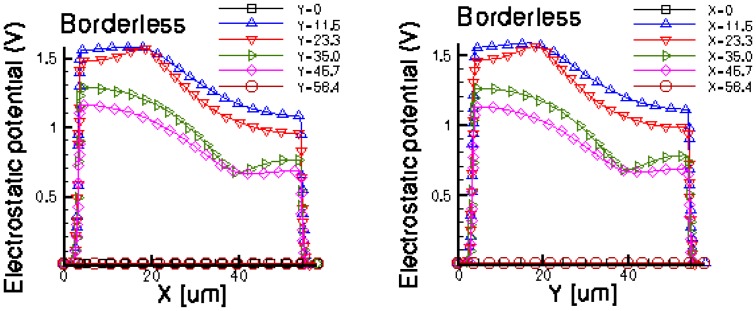
Electrostatic potential (V) in orthogonal cuts on Oy and Ox for optimum and borderless structures.

Figures 14 and 15 present the electrostatic potential at the surface of the devices (Z = 0) for all simulated cells in the case of 1 mA biasing current and magnetic field B = 0.5 T with orthogonal cuts on Oy and Ox respectively, applied on the 3D Hall Effect devices structures, as presented in Figures 8–12. Therefore, the Ox and Oy are the regular axes of the three-dimensional structures, as presented in Figures 2–6, corresponding to the sides of the p-substrate onto which the devices are built. Among the cross-like structures, the maximum electrostatic potential is on the biasing electrode a. It is equal to 2.6 V for the basic cell, 2.7 V for the L and XL cells respectively. As the theory indicates, the absolute sensitivity of Hall cells fabricated under the same technological process is only dependent on the geometrical correction factor G which in its turn is directly proportional to L/W. Therefore, for shapes with the same length to width ratio L/W, it is expected to have the same sensitivity.

**Figure 16.**
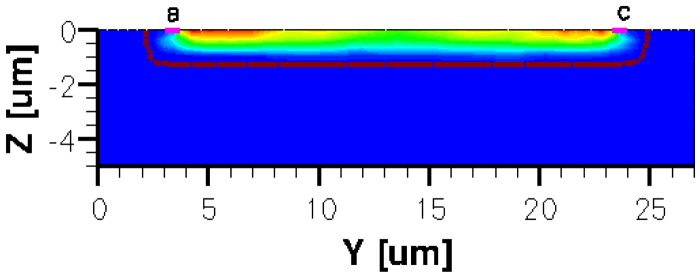
Conduction current density for basic cell with emphasis on the biasing contacts *a* and c.

**Figure 17.**
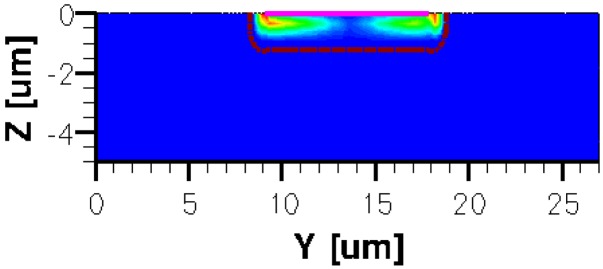
Conduction current density for basic cell (X = 3.25).

**Figure 18.**
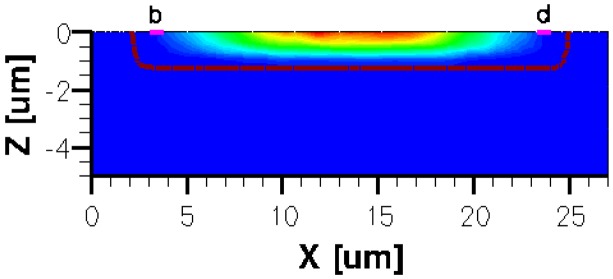
Conduction current density for basic cell, with emphasis on the sensing contacts *b* and *d* (Y = 12.9).

The same type of graphs is also investigated for the optimum and borderless cell respectively. The peak of the electrostatic potential is 1.8 V for the borderless cell and 2.3 V for the optimum cell. From these graphs we can also deduct the length of the electric contacts, for optimum and borderless cells as the electrostatic potential is constant in that region. On the Ox cuts of L, XL, basic cells, the descent from the peak is not always a straight line due to the non-homogeneity in material mobility, conductivity, sheet resistance, *etc.* In Figures 16–18, different cuts were performed on the three-dimensional simulated structures in order to reveal the conduction current density, with accent on the biasing contacts (*a* and *c*) and sensing contacts (*b* and *d*) respectively.

### 3.3. Absolute, Current-Related and Voltage-Related Sensitivities of the Simulated Hall Cells

**Figure 19.**
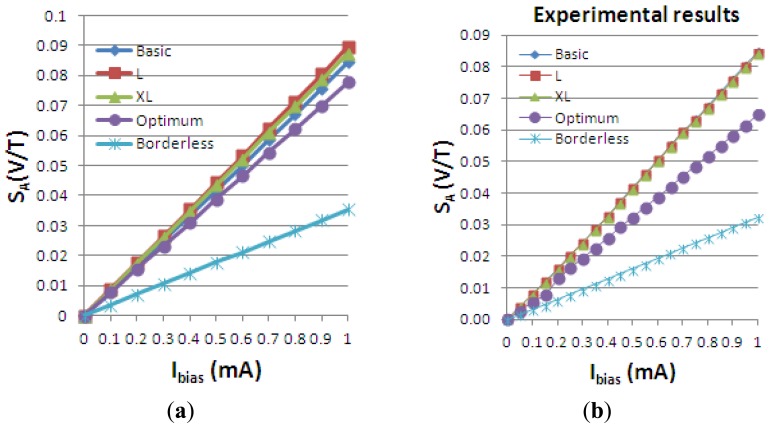
Hall Effect devices absolute sensitivity *vs. I_bias_*, as simulated (**a**) and as measured (**b**).

**Figure 20.**
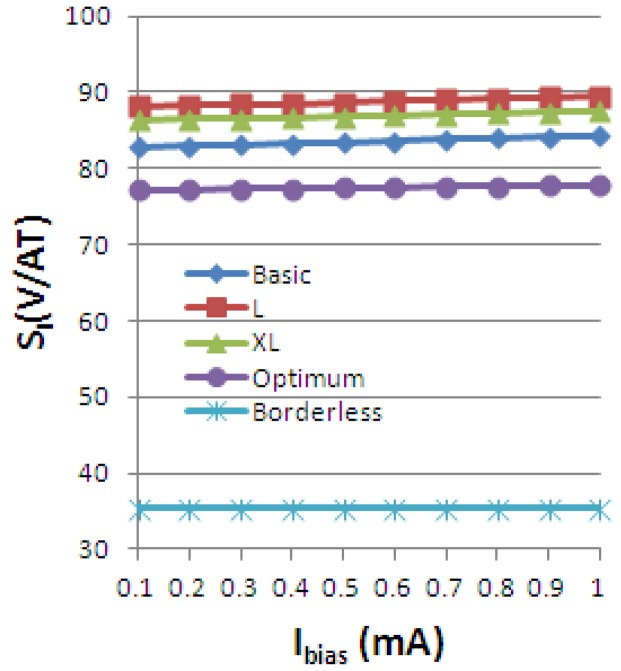
Current-related sensitivity of the simulated Hall Effect devices.

**Figure 21.**
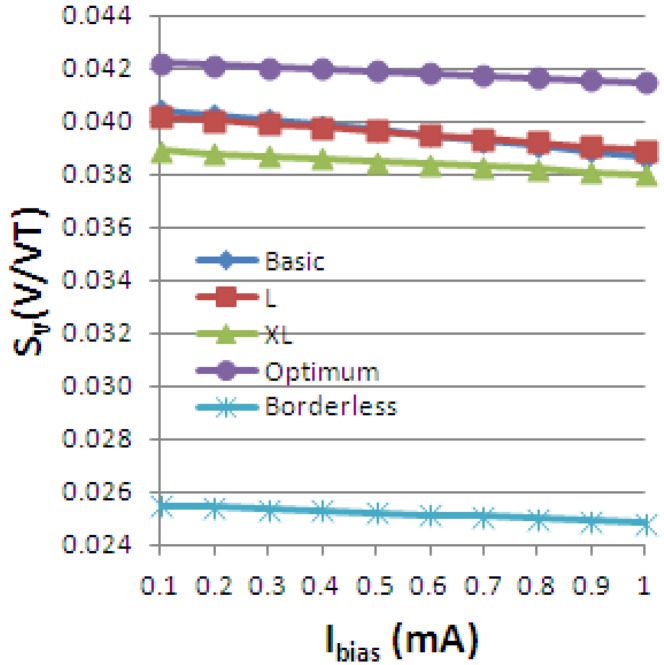
Voltage-related sensitivity of the simulated Hall Effect devices.

Using the definitions for the absolute, current-related and voltage-related sensitivities in Equation (4), we obtain the following graphs, Figures 19–21, plotted *vs.* the biasing current, for a magnetic field intensity B = 0.5 T. Among the analyzed cells, for a current polarization, the XL cell displayed the highest absolute sensitivity and therefore current-related sensitivity. Nevertheless, for a voltage polarization of the devices, the optimum cell appears to be the best candidate as it has the maximum voltage-related sensitivity. In Figure 21, the voltage-related sensitivity of the borderless cell is smaller by a factor of 1.7 with respect to the optimum cell. The numerical results reported for Hall voltage and sensitivity are in good agreement with the experimental results [3], up to the extents of precise reproduction by simulation of the real structures tested. In Figure 19, both simulated and experimental results are presented regarding Hall Effect devices absolute sensitivity *vs.* the biasing current.

The current-related sensitivity is increasing with the biasing current, while the voltage-related sensitivity is decreasing with the biasing current. The explanation of the latter mechanism is the fact that S_V_ can be rewritten as the ratio of the current-related sensitivity to the input resistance *R* and the denominator *R* increases more rapidly with the current than the numerator *S_A_*.

The selection process of the best Hall Effect device is based on analyzing several parameters behavior such as sensitivity, offset, dissipated power and Silicon surface. By consequence, it is advised to inspect a more complex cost function. There are circuit methods such as the current spinning technique to reduce the offset and the Silicon surface can be traded by the designer for a high sensitivity. Therefore, obtaining the highest sensitivity with a good tradeoff with offset and power dissipated seems to be prevalent.

The dissipated power was calculated within each structure. The ratio of the absolute sensitivity to the power dissipated within the device was also investigated. Even though the relation (6) for power-related sensitivity is not a standalone equation and can be deducted from Equation (4), it is nevertheless worthwhile to investigate it:

(6) $$S_{P}=\frac{S_{A}}{P_{\text{dissipated}}}\;\left[\frac{V}{WT}\right]$$

**Figure 22.**
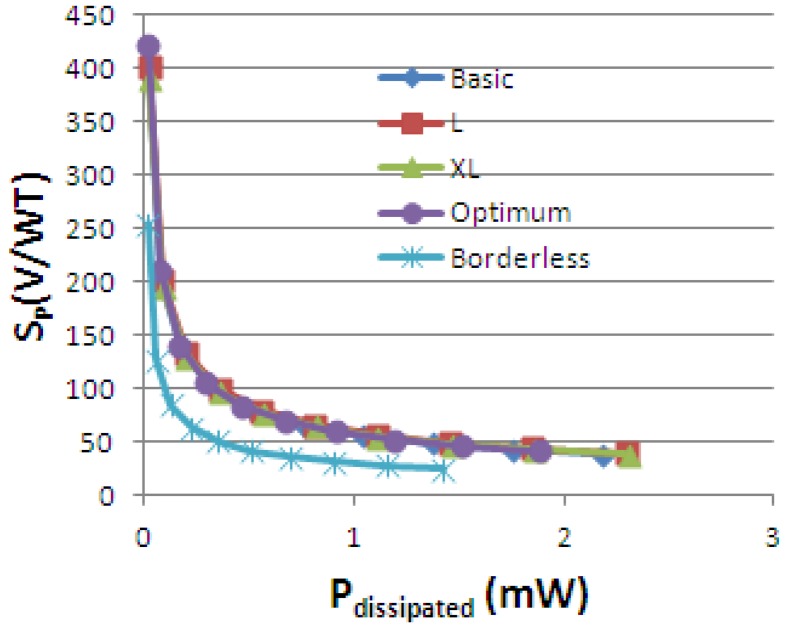
Power-related sensitivity *vs.* the dissipated power for the simulated Hall Effect devices.

The variation of power-related sensitivity *S_P_vs.* the dissipated power is presented in Figure 22. For *I_bias_* = 1 mA, the maximum dissipated power is 2.18 mW for the basic cell, 2.3 mV for L and XL cells, 1.87 mW for the optimum cell and 1.42 mW for the borderless cell respectively. Nevertheless, even if the lowest dissipated power is obtained for low current, we cannot work in that region because the noise is prevalent and the signal to noise ratio (SNR) is too low.

We can note that the Greek-like cells and the optimum cell have almost the same power-related sensitivity, with a slightly higher value for the optimum cell. We can observe that for this particular figure of merit, the geometry has less importance, with the best performance belonging to the optimum cell. There is an improvement of almost 10% of the optimum cell with respect to XL structure for constant current of 1 mA. From this perspective, the optimum cell seems like a good candidate.

The above discussion on different types of sensitivities leads to the conclusion that the Hall device polarization is important and dictates which shape should be chosen in order to guarantee the best performance.

### 3.4. Simulated Hall Cells Induced Offset Analysis

**Figure 23.**
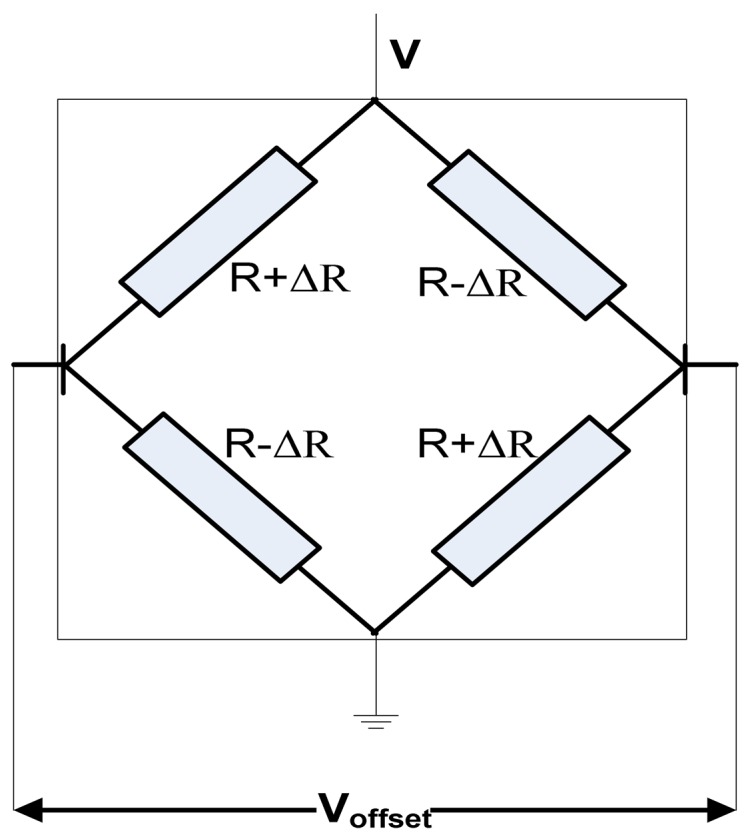
The bridge circuit model of a Hall cell.

Offset voltage can be generated by imperfections in the fabrication process, misalignment of contacts, non-uniformity of material resistivity and thickness, mechanical stress in combination with the piezoresistance effect [6]. According to Popovic's book, all these causes can be represented using the general bridge circuit model of a Hall plate, based on four resistances on each branch [6], as shown in Figure 23. For an ideal cell, the four resistors on the branches are equal, according to the classical Hall device theory. However, any little variation *ΔR* of the branch resistance would produce an asymmetry of the bridge and the result will be *V_offset_*.

Therefore, the offset voltage caused by an asymmetry of the bridge is given by:

(7) $$V_{\text{offset}}=\frac{\Delta R}{R}\;V_{in}$$

where *V_in_* is the input voltage and *R* is the branch resistance.

**Figure 24.**
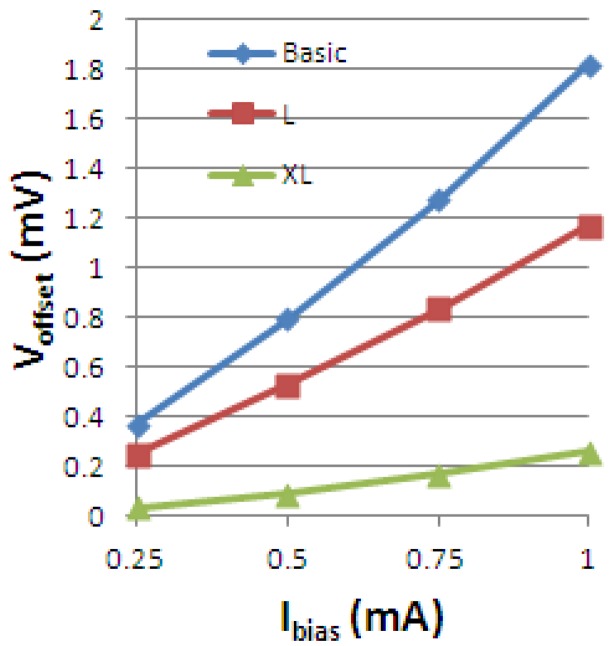
The measured single phase offset *vs.* biasing current for the integrated cross-like Hall Effect devices.

Previous measurements performed on the Hall Effect devices for offset evaluation released information about how this quantity changes with the shape. Numerical values of the offset for the cross-like integrated cells are displayed in Figure 24 below. We mention that this is the measured single phase offset, so it is before averaging it on several phases.

**Figure 25.**
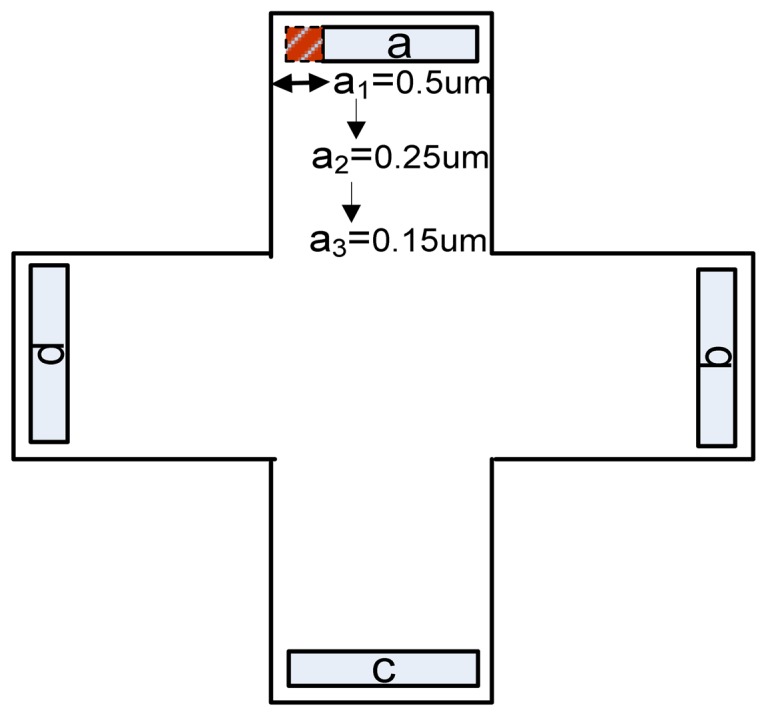
The induced asymmetry on the biasing contact *a* for cross-like Hall cells.

An analysis of the artificial offset is now intended to see how an induced asymmetry can influence the offset and finally find the shape to guarantee the lowest value of this quantity. At this point, we mainly focus on the offset that might be created by a misalignment in the sensors shape. The Hall devices are equipped with two biasing contacts (*a* and *c*) and two sensing contacts (*b* and *d*). The following figure presents how the asymmetry was induced, for cross-like Hall cells, in the first instance on the biasing contact *a* by removing 0.5 μm (asymmetry 1), 0.25 μm (asymmetry 2), 0.15 μm (asymmetry 3), respectively (Figure 25).

As the offset is a random process, a possible location was presumed on the contact *a.* However, other asymmetries can be induced on the rest of the contacts, as desired.

**Figure 26.**
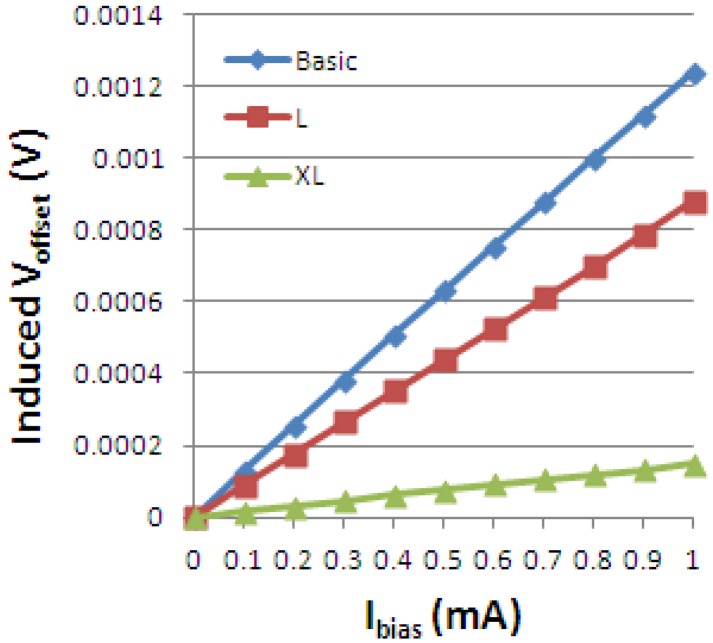
Simulated artificial offset of the cross structures induced by an *a_1_* asymmetry.

In the present work, simulated artificial offsets induced by small Ox-axis displacements of 0.5, 0.25 or 0.15 μm on the biasing contact *a* have been analyzed. The following effect of a 0.5 μm displacement on the cross-like Hall cells offset is presented in Figure 26. As we are aiming to emphasize the information related to the induced offset only, the numerical offset was subtracted from the total offset.

For the XL cell, the influence of the mismatch on the offset was the least. It seems that by increasing the dimensions of a cell we will be diminishing any errors that might appear on the borders and therefore minimizing the offset. This fact was also analyzed in [5] by the authors and experimental data confirms this assumption. Nevertheless the offset is a random process. For correctly characterizing it we need a statistics that could provide systematization of the offset generation possible causes and to quantitatively correlate these influences with the effect on the offset value. This is envisioned in future papers.

### 3.5. Simulated Hall Cells Performance Summary

The performances of all the analyzed Hall Effect sensors, including Hall voltage, current-related sensitivity, power-related sensitivity and dissipated power are summarized in the Table 3 for a biasing current of 1 mA. This is simulated data, but the experimental results previously presented in the paper support the general conclusions regarding the Hall Effect sensors performance.

## 4. Conclusions

The influence of the shape, dimensions, offset on the Hall Effect sensors performance was analyzed using 3D physical simulations capable of considering the magnetic field influence on semiconductors.

To this purpose, five different Hall Effect sensors of a certain technological CMOS fabrication process were modeled. The Hall Effect sensor configurations were simulated and evaluated for Hall voltage, absolute, current-related, voltage-related, power-related sensitivities and offset.

The estimations of these important parameters finally allow choosing the best shape depending on the device polarization used in the circuit and the figures of merit priority. In particular, the simulation and experimental results are in good agreement.

The simulation procedure guides the designer in accurately modeling and characterizing specific magnetic sensor shapes of a certain fabrication technology. Estimating their Hall voltage, sensitivity will aid in choosing the Hall cell optimum fabrication process, shape, dimensions and polarization in terms of the performances envisaged to be achieved.

Future investigations on the causes of the offset and a systematic approach that would correlate the causes with quantification in the offset value are envisaged to be performed in future articles.

## Figures and Tables

**Table 1.** Geometrical parameters of the simulated Hall devices.

**Type of Hall cell**	**L (μm)**	**W (μm)**	**s (μm)**	**Volume (μm**^**3**^**)**
***Basic***	21.6	9.5	8.8	3,645
***L***	32.4	14.25	13.55	7,144.2
***XL***	43.2	19	18.3	11,809.8
***Borderless***	50	50	2.3	16,820
***Optimum***	54	54	4.7	17,052.8

**Table 2.** Simulated Hall Effect devices resistance.

**R (kΩ)**	**I = 0.3 mA**	**I = 0.5 mA**	**I = 1 mA**
***Basic***	2.073	2.102	2.181
***L***	2.212	2.235	2.298
***XL***	2.236	2.254	2.302
***Optimum***	1.837	1.847	1.874
***Borderless***	1.393	1.400	1.422

**Table 3.** Simulated Hall Effect devices performance summary.

**Type of Hall cell**	**V**_**HALL**_**(mV)**	**S**_**I**_**(V/AT)**	**S**_**P**_**(V/WT)**	**P**_**dissipated**_**(mW)**
***Basic***	42.18	84.360	38.644	2.182
***L***	44.75	89.500	38.907	2.300
***XL***	43.74	87.480	37.973	2.303
***Optimum***	38.91	77.830	41.482	1.876
***Borderless***	17.70	35.390	24.840	1.424
